# Functional effects of muscle PGC-1alpha in aged animals

**DOI:** 10.1186/s13395-020-00231-8

**Published:** 2020-05-06

**Authors:** Steven Yang, Emanuele Loro, Shogo Wada, Boa Kim, Wei-Ju Tseng, Kristina Li, Tejvir S. Khurana, Zoltan Arany

**Affiliations:** 1 Department of Medicine, Perelman School of Medicine, University of Pennsylvania, Philadelphia, PA USA; 2 Department of Physiology and Pennsylvania Muscle Institute, Perelman School of Medicine, University of Pennsylvania, Philadelphia, PA USA; 3 Department of Orthopaedic Surgery, Perelman School of Medicine, University of Pennsylvania, Philadelphia, PA USA

## Abstract

PGC-1 (peroxisome-proliferator-activated receptor-γ coactivator-1) alpha is a potent transcriptional coactivator that coordinates the activation of numerous metabolic processes. Exercise strongly induces PGC-1alpha expression in muscle, and overexpression of PGC-1alpha in skeletal muscle activates mitochondrial oxidative metabolism and neovascularization, leading to markedly increased endurance. In light of these findings, PGC-1alpha has been proposed to protect from age-associated sarcopenia, bone loss, and whole-body metabolic dysfunction, although these findings have been controversial. We therefore comprehensively evaluated muscle and whole-body function and metabolism in 24-month-old transgenic mice that over-express PGC-1alpha in skeletal muscle. We find that the powerful effects of PGC-1alpha on promoting muscle oxidative capacity and protection from muscle fatigability persist in aged animals, although at the expense of muscle strength. However, skeletal muscle PGC-1alpha does not prevent bone loss and in fact accentuates it, nor does it have long-term benefit on whole-body metabolic composition or insulin sensitivity. Protection from sarcopenia is seen in male animals with overexpression of PGC-1alpha in skeletal muscle but not in female animals. In summary, muscle-specific expression of PGC-1alpha into old age has beneficial effects on muscle fatigability and may protect from sarcopenia in males, but does not improve whole-body metabolism and appears to worsen age-related trabecular bone loss.

## Background

Metabolic homeostasis requires a complex network of transcriptional programs. PGC-1 (peroxisome-proliferator-activated receptor-γ coactivator-1) alpha is a potent transcriptional coactivator that regulates a large number of nuclear-encoded genes [1–3], which, in turn, modulate numerous metabolic processes. In most cells and tissues, PGC-1alpha drives activation of mitochondrial biogenesis. In addition, PGC-1alpha promotes brown fat differentiation and thermogenesis [4], hepatic gluconeogenesis [5], cardiac homeostasis [6], and axonal integrity in the brain [7].

PGC-1alpha has also been widely studied in skeletal muscle. Exercise strongly induces muscle PGC-1alpha in both humans and rodents [8–10], and overexpression of PGC-1alpha in skeletal muscle activates mitochondrial oxidative metabolism [11], leading to markedly increased endurance [12]. Skeletal muscle PGC-1alpha also induces neovascularization and is required for exercise-induced angiogenesis [13–15], and protects against muscle dystrophy in ways not well understood [16].

These remarkable benefits of PGC-1alpha expression in skeletal muscle have raised the possibility that PGC-1alpha may protect against age-associated functional decline of muscle. A widely cited report indicated that PGC-1alpha protects against sarcopenia and loss of bone mineral density in aged mice, as well as improves whole-body insulin sensitivity [17], but this report has since been retracted, leaving these questions unanswered. A more recent report showed that overexpression of skeletal muscle PGC-1alpha improves muscle endurance, motor coordination, and balance in aged animals [18], but the effects of skeletal muscle PGC-1alpha overexpression on other parameters, such as muscle contractility, bone structural integrity and whole-body metabolism, were not investigated.

To resolve these unanswered questions, we endeavored to comprehensively evaluate muscle and whole-body function and metabolism in 24-month-old, male and female mice over-expressing PGC-1alpha in skeletal muscle.

## Methods

### Quantitative RT-PCR (qPCR)

Snap frozen quadriceps were lysed using TRIzol (Invitrogen 15596026). mRNA was then extracted with chloroform and reverse-transcribed using the High Capacity cDNA Reverse Transcription Kit (Applied Biosystems 4368813). qPCR was performed using Excella SYBR MasterMix (WorldWide Life Sciences Division 61071093). All qPCR data were normalized to expression of housekeeping genes *Actb*, *Hprt*, and *Tbp*.

### Western blots

Protein was isolated from snap frozen quadriceps in RIPA Lysis Buffer (VWR 97063-270) supplemented with protease inhibitor (11836153001 ROCHE) and phosphatase inhibitor (PHOSS-RO ROCHE). Western blot was performed using antibodies against total OXPHOS (abcam ab110413), HSP90 (CST 4874S), pAKT (Ser473) (CST 9271S), and pan AKT (CST 4691S). Ten mircograms of protein/sample was loaded per well.

### Cryosectioning

Tibialis anterior and EDL muscles were isolated and embedded in OCT freezing matrix and frozen in isopentane cooled in liquid nitrogen. Ten micron-thick cross sections were then mounted on microscope slides.

### Capillary staining

Tibialis anterior cryosections were fixed in 4% paraformaldehyde for 30 min. Slides were then permeabilized in 0.5% Triton X-100 solution and then blocked in 5% BSA solution for 1 h. The slides were then incubated with CD31 antibody (Millipore MAB1398Z) overnight at 4 °C. The slides were then incubated with secondary antibodies and WGA (ThermoFisher W6748) for 4 h and mounted with VECTASHIELD mounting media with DAPI.

### Ex vivo muscle contractility analysis

Muscle physiological analysis was performed on isolated EDL and soleus muscles using an Aurora Mouse 1200A System equipped with Dynamic Muscle Control v.5.415 software. EDL muscles were dissected and analyzed in constantly oxygenated Ringer’s solution (100 mM NaCl, 4.7 mM KCl, 3.4 mM CaCl_2_, 1.2 mM KH_2_PO_4_, 1.2 mM MgSO_4_, 25 mM HEPES, 5.5 mM D-glucose) at 24 °C. The twitch stimulation protocol applied was a single stimulus with a duration of 0.2 ms. Muscle length was adjusted to obtain the maximal twitch response, and this length was measured and recorded as optimal length (*L*_0_). For measuring tetanic maximal force generation, the stimulus was repeated at a frequency of 120 Hz (EDL) or 80 Hz (soleus) for 500 ms. Five minutes were allowed between two tetanic contractions to ensure muscle recovery. For induction of fatigue, 5 min after the last maximal tetanic contraction, muscles were stimulated every second for 8 min using 40-Hz pulses lasting 330 ms. Muscle cross-sectional area (CSA) of EDL muscles was calculated dividing the muscle mass by the product of the muscle density coefficient (1.06 g/cm^3^), muscle *L*_0_, and the fiber length coefficient (0.45 for EDL, 0.69 for soleus). Specific force was determined by normalizing maximum isometric tetanic force to CSA.

### Bone micro-computed tomography (μCT)

Trabecular (1.2 mm region proximal to growth plate, 6 μm isotropic voxels) and cortical (0.3 mm at 50% length, 6 μm isotropic voxels) bone parameters were measured in dissected femurs using μCT (microCT 35, ScancoMedical AG, Brüttisellen, Switzerland). Manufacturer-provided software for 3D standard microstructural analysis was used to calculate trabecular bone mineral density (BMD), bone volume fraction (BV/TV), trabecular thickness (Tb.Th), trabecular separation (Tb.Sp), trabecular number (Tb.N), structure model index (SMI), connectivity density (Tb.Conn.D), degree of anisotropy (DA), bone surface (BS), cortical tissue mineral density (Ct.TMD), cortical area (Ct.Ar), and cortical bone thickness (Ct.Th).

### Glucose tolerance test (GTT)

Animals were fasted overnight (7 pm–9 am). The next morning, following measurement of fasting glucose, mice were gavaged with D-glucose solution (2 g/kg), and blood glucose levels were measured at 15, 30, 60, and 120 min.

### Physiology assay protocols

Body composition of animals was analyzed by NMR at Mouse Phenotyping, Physiology and Metabolism Core, Perelman School of Medicine, University of Pennsylvania. Then, mice were individually housed in the cages, and their metabolic physiology (VO2, VCO2 and RER) was monitored by comprehensive laboratory animal monitoring system (CLAMS) at the Mouse Phenotyping, Physiology and Metabolism Core, Perelman School of Medicine, University of Pennsylvania.

### Mouse models

All mouse experiments were performed according to procedures approved by the University of Pennsylvania Institutional Animal Care and Use Committees. MCKa mice were described previously [11]. All mice were in a C57BL/6 background, maintained in regular 12 hr light/dark cycles, kept in cages of 3–5 animals per cage, separating males from females, and fed regular chow ad libitum. Males and females were used experimentally as indicated in figures.

### Statistical analyses

*P* values were calculated using the two-tailed student *t* test. For statistical comparisons between study groups, two-way ANOVA was used. *P* < 0.05 was considered to be statistically significant. Data are displayed as mean ± standard error.

## Results

**Fig. 1:**
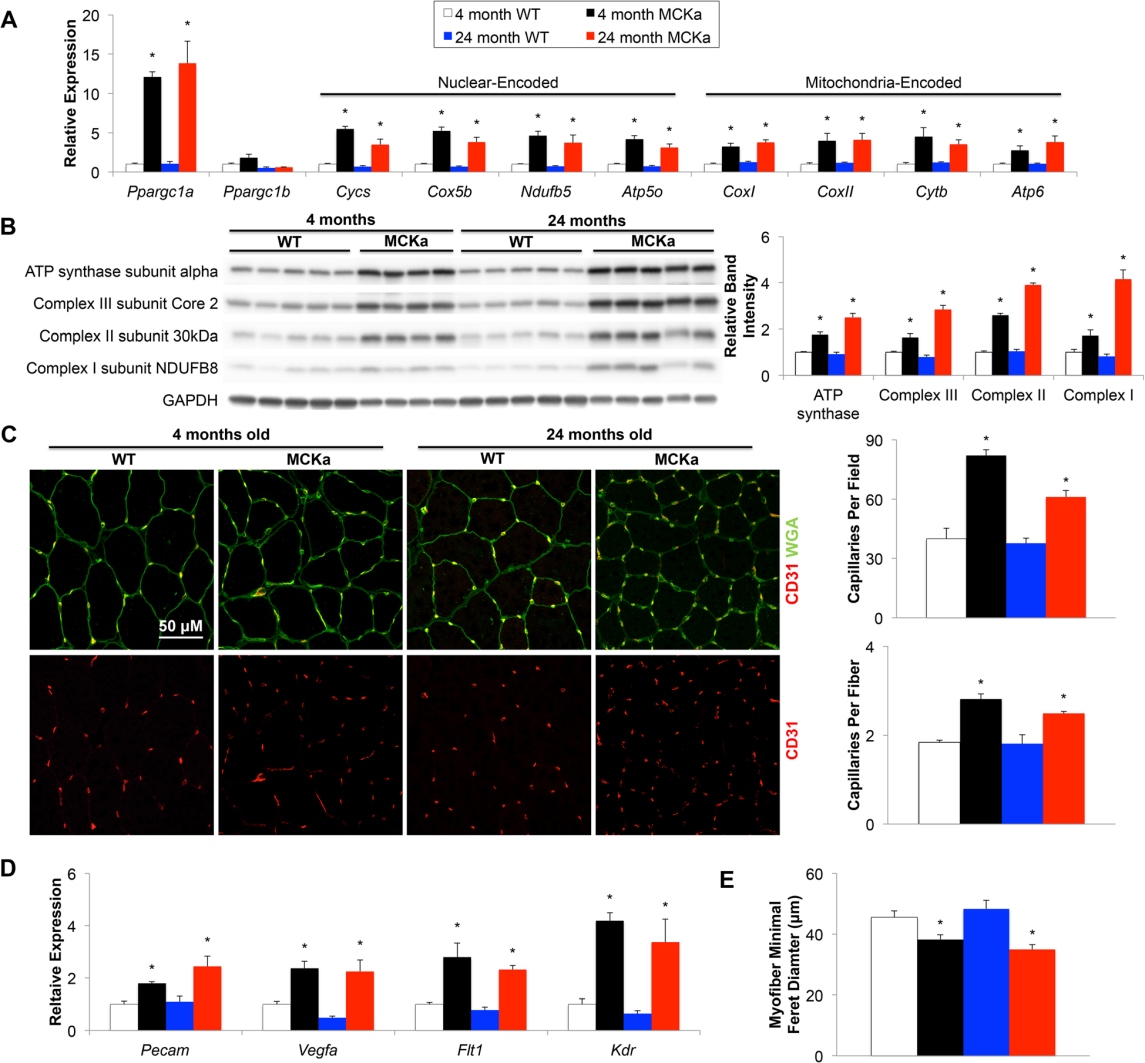
PGC-1alpha promotes mitochondrial biogenesis and angiogenesis in 24-month-old mice. **a** Quantitative real time-polymerase chain Rreaction (qPCR) of PGC1 coactivators and components of the electron transport chain on quadriceps isolated from 4-month or 24-month-old wildtype and MCKa mice. **b** Western blots for oxidative phosphorylation proteins in quadriceps (quantification is normalized to total protein using amido black). **c** CD31 immunohistochemistry on tibialis anterior cryosections. **d** qPCR for endothelial markers and angiogenesis genes in quadriceps. **e** Myofiber minimal Feret diameter in tibialis anterior cryosections [*n* = 5, 4, 5, 5 for WT (4 mo), MCKa (4 mo), WT (24 mo), and MCKa (24 mo) respectively]. *P < 0.05 compared to control

We started by investigating whether the process of aging alters any of the previously characterized functions of PGC-1alpha in skeletal muscle. We aged to 24 months mice with transgenic expression of PGC-1alpha under control of the muscle-specific muscle creatine kinase (MCK) promoter (MCKa mice) [11], and compared them both to littermate controls and to analogous 4-month-old groups. The PGC-1alpha transgene was equivalently expressed in young and old animals, as determined by qPCR (Fig. 1a). We first looked at mitochondrial biogenesis and genes of oxidative phosphorylation (OXPHOS), well known to be induced by PGC-1alpha in skeletal muscle [11]. Western blot and qPCR analyses of skeletal muscle lysates from 24-month-old animals revealed markedly elevated expression of OXPHOS genes and protein in MCKa animals compared to wildtype littermate controls (Fig. 1a, b). This increase was equivalent in young (4 month) and old (24 month) animals, indicating that the mitochondrial biogenic program of skeletal muscle PGC-1alpha is maintained in aged animals. PGC-1alpha also potently drives angiogenesis in skeletal muscle [13, 14]. We compared capillary density between skeletal muscle of transgenic and wildtype animals in both young and old age. CD31 staining of tibialis anterior cryosections from young and old animals revealed that MCKa animals have increased capillary density, and that this increase is maintained in old age (Fig. 1c). mRNA levels of endothelial cell markers and angiogenesis signaling proteins were also significantly elevated in the transgenic animals compared to controls, and this increase was equivalent in young and old animals (Fig. 1d). Finally, consistent with PGC-1alpha-mediated transformation of larger glycolytic fibers to smaller oxidative fibers, myofiber size is decreased in transgenic animals (Fig. 1e). Aged animals thus retain the capacity to increase mitochondrial biogenesis and angiogenesis in response to PGC-1alpha induction.

**Fig. 2:**
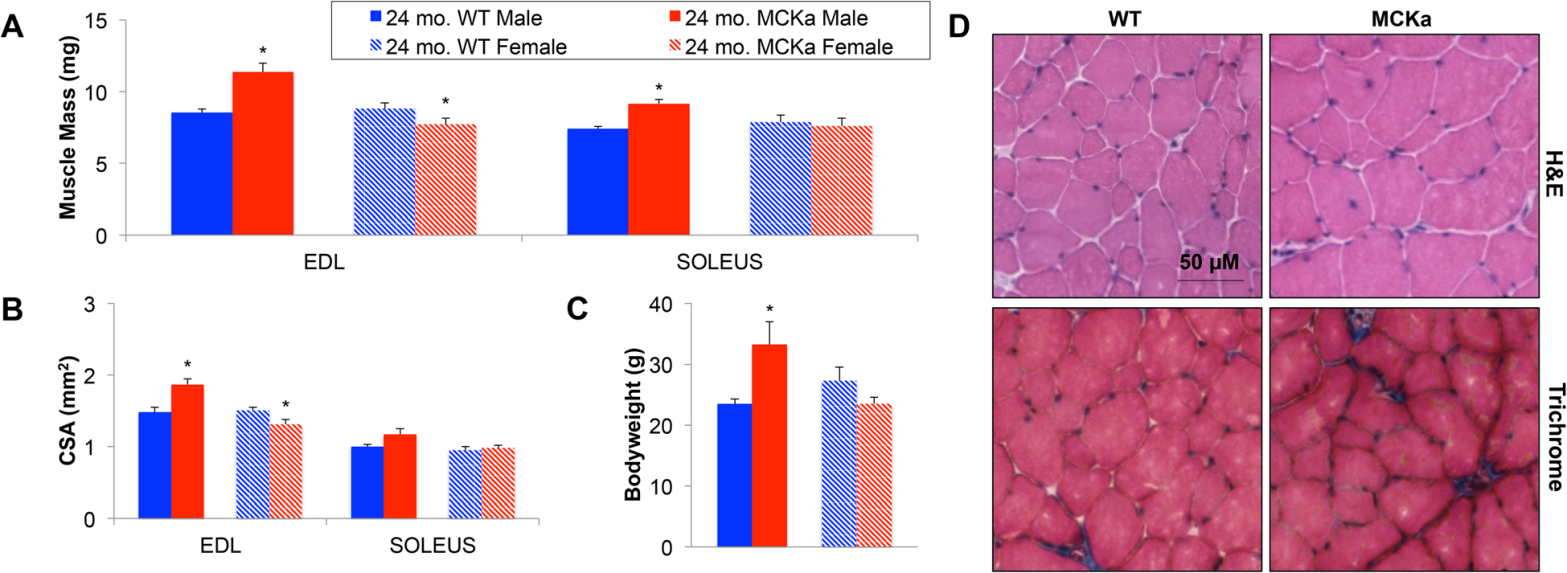
Muscle mass and muscle cross-sectional area are conserved in male 24-month-old MCKa mice. **a** Muscle mass of isolated extensor digitorum longus (EDL) and soleus muscles. **b** Cross-sectional area of isolated EDL and soleus muscles. **c** Bodyweight of mice. **d** Haemotoxylin and eosin (H&E) and Masson’s trichrome staining of EDL cryosections from 24-month-old mice [*n* = 5, 5, 5, 4 for 24-month-old WT (male), MCKa (male), WT (female), and MCKa (female) respectively]. *P < 0.05 compared to control

Elevation of PGC-1alpha in skeletal muscle has been previously suggested to protect from age-associated muscle mass loss (sarcopenia) but, as noted above, that report has been retracted [17]. We find that the mass of glycolytic extensor digitorum longus (EDL) and of oxidative soleus muscles (Fig. 2a) and the muscle cross-sectional areas in the same muscles (Fig. 2b) are higher in 24-month-old male MCKa animals, compared to littermate controls, indicating that indeed MCKa may protect from sarcopenia. It is important to note, however, that the MCKa mice also had higher bodyweights than littermate controls (Fig. 2c), suggesting that the larger muscle may not be a cell autonomous effect. Interestingly, we did not observe protection from sarcopenia (Fig. 2a, b) or higher body weights (Fig. 2c) in the aged females, suggesting a possible sexual dimorphic effect of skeletal muscle PGC-1alpha in the protection from sarcopenia. Finally, we observed no differences in central nucleation, or evidence of fibrosis, between age-matched MCKa and wildtype animals (Fig. 2d).

**Fig. 3:**
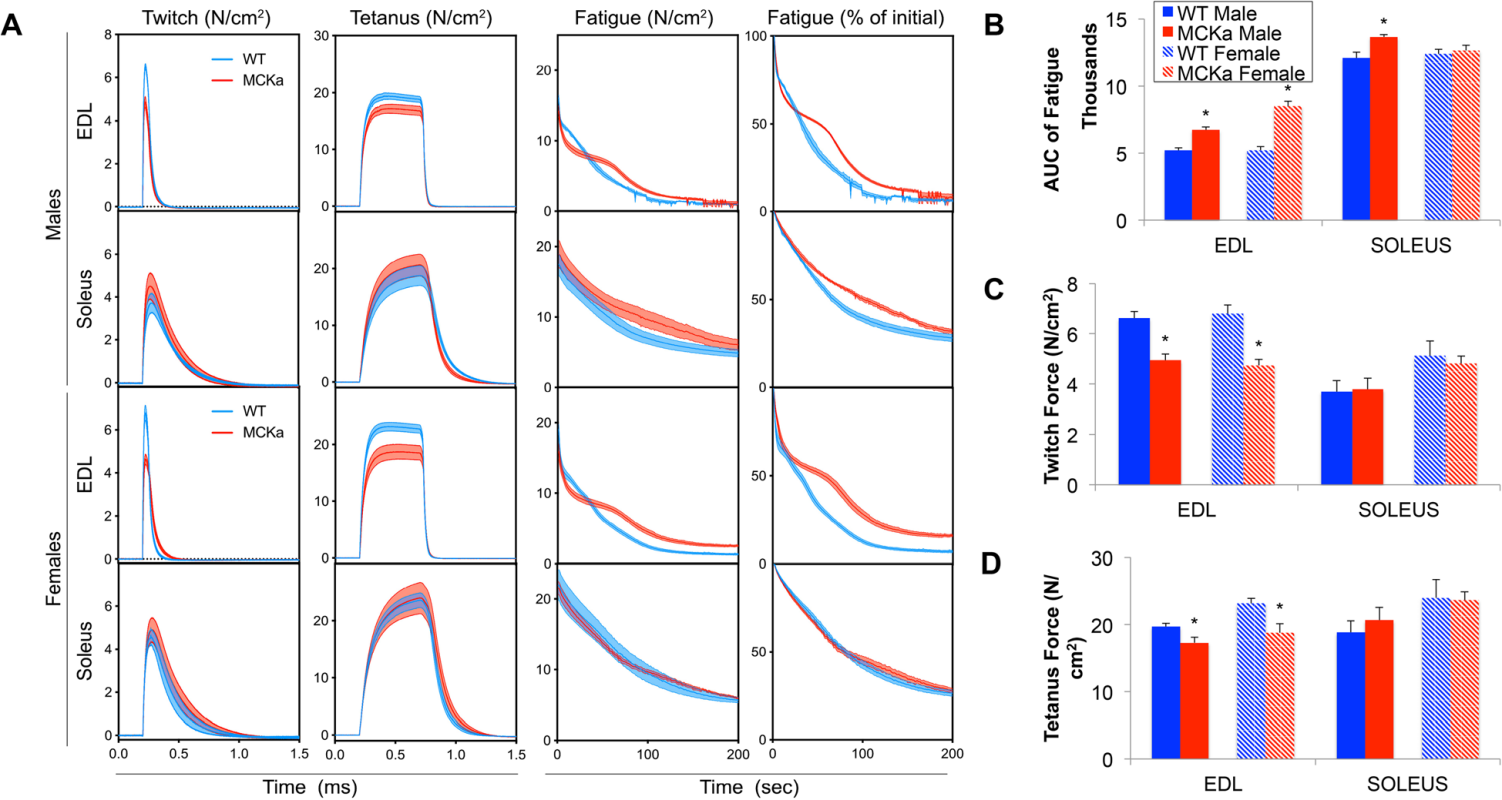
Twenty-four-month-old MCKa animals have less fatigable muscles at the expense of decreased strength (twitch and tetanus force). **a** Traces of muscle contraction in 24-month-old mice during twitch, tetanus, and fatigue protocols. **b** AUC of fatigue curves. **c** Maximum twitch force generated. **d** Maximum tetanus force generated [*n* = 5, 4, 5, 4 for 24-month-old WT (male), MCKa (male), WT (female), and MCKa (female) respectively]. *P < 0.05 compared to control

We next evaluated the functional effects of elevated PGC-1alpha on skeletal muscle in aged animals. We measured *ex vivo* both the contractile properties and the muscle fatigability of EDL and soleus muscles from 24-month-old mice. We found that in both males and females, EDL muscles from MCKa mice were markedly less fatigable than littermate controls (Fig. 3a and b), at the expense of significantly reduced twitch (Fig. 3a and c) and tetanic force (Fig. 3a and d). These differences were largely observed only in the fast-glycolytic EDL, whereas little difference was observed in the slow-oxidative soleus. PGC-1alpha thus can reduce fatigability in aged animals, as it does in young animals, though at the expense of muscle strength [12].

**Fig. 4:**
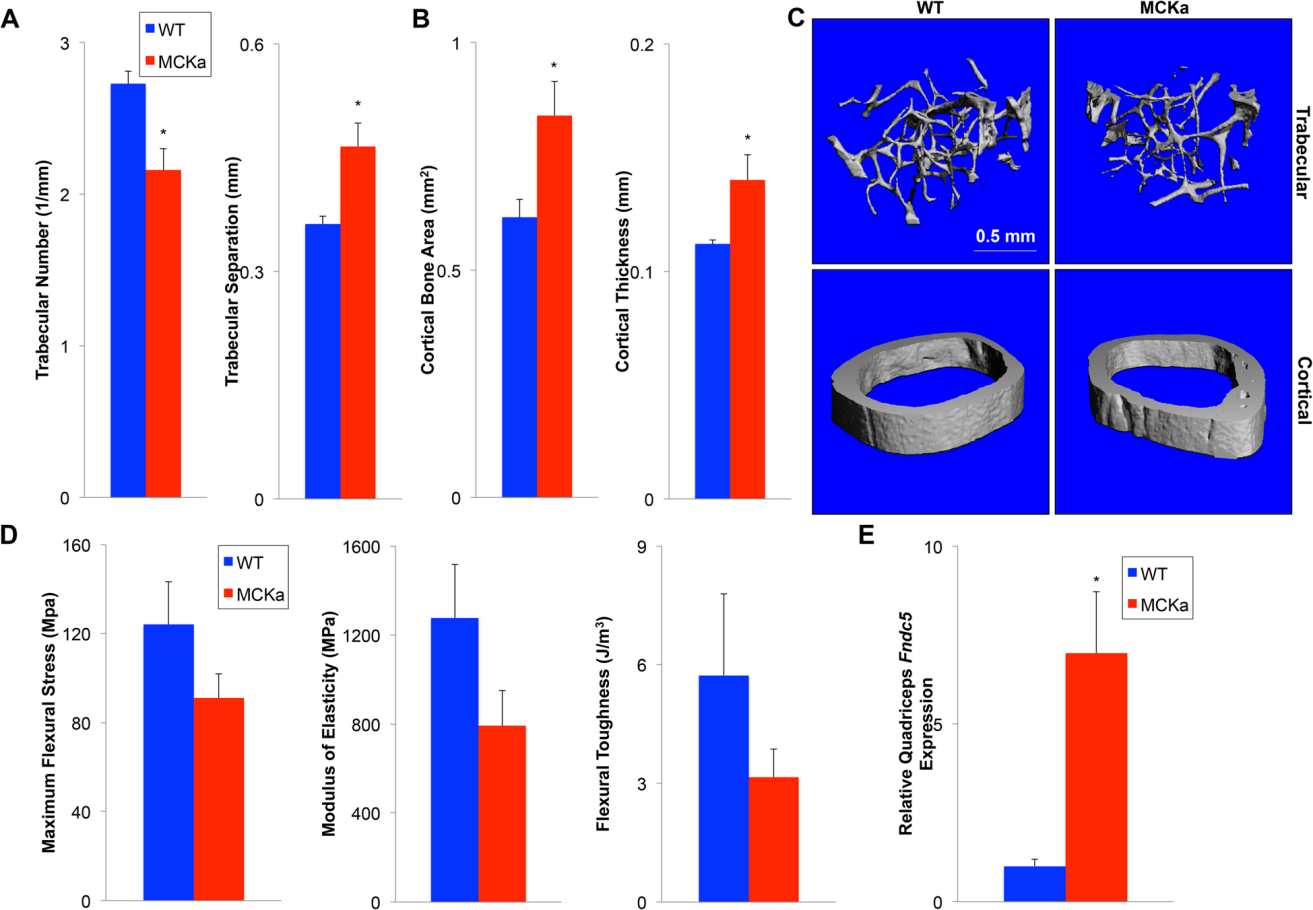
Twenty-four-month-old MCKa animals have worsened age-associated trabecular bone loss. **a** Quantification of average trabecular number and trabecular separation in isolated femurs from 24-month-old male mice. **b** Quantification of average cortical bone area and cortical bone thickness in isolated femurs from 24-month-old male mice. **c** Micro-computed topography (micro-CT)-generated 3-dimensional scans of trabecular and cortical bone in isolated femurs. **d** Maximum flexural stress, elasticity, and flexural roughness of isolated femurs from 24-month-old male mice. **e** qPCR of *Fndc5* in quadriceps isolated from 24-month-old male mice [*n* = 5, 5 for 24-month-old WT and MCKa]. *P < 0.05 compared to control

Expression of PGC-1alpha in skeletal muscle was also proposed to protect from age-associated bone loss [17]. We therefore used microCT scans to generate 3D images of both the cortical and trabecular bone in femurs dissected from aged MCKa and control animals. We observed that the transgenic animals have increased cortical bone area and cortical bone thickness (Fig. 4b, c), while trabecular number is decreased in the transgenic animals compared to wildtype controls (Fig. 4a, c). Loss of trabecular bone is a common indicator of age-associated bone loss [19, 20]. Consistent with this, we find that femurs from aged MCKa animals trend towards having remarkably lower elasticity, flexural toughness, and maximum flexural stress during a 3-point bending test (Fig. 4d). Additionally, we find that *Fndc5* mRNA expression is elevated in the skeletal muscle of MCKa animals (Fig. 4e). FNDC5, a target of PGC-1alpha, is required for trabecular bone loss following ovariectomy [21, 22]. These data thus not only suggest that induction of PGC-1alpha in skeletal muscle that does not ameliorate age-associated trabecular bone loss, but it may actually worsen it.

**Fig. 5:**
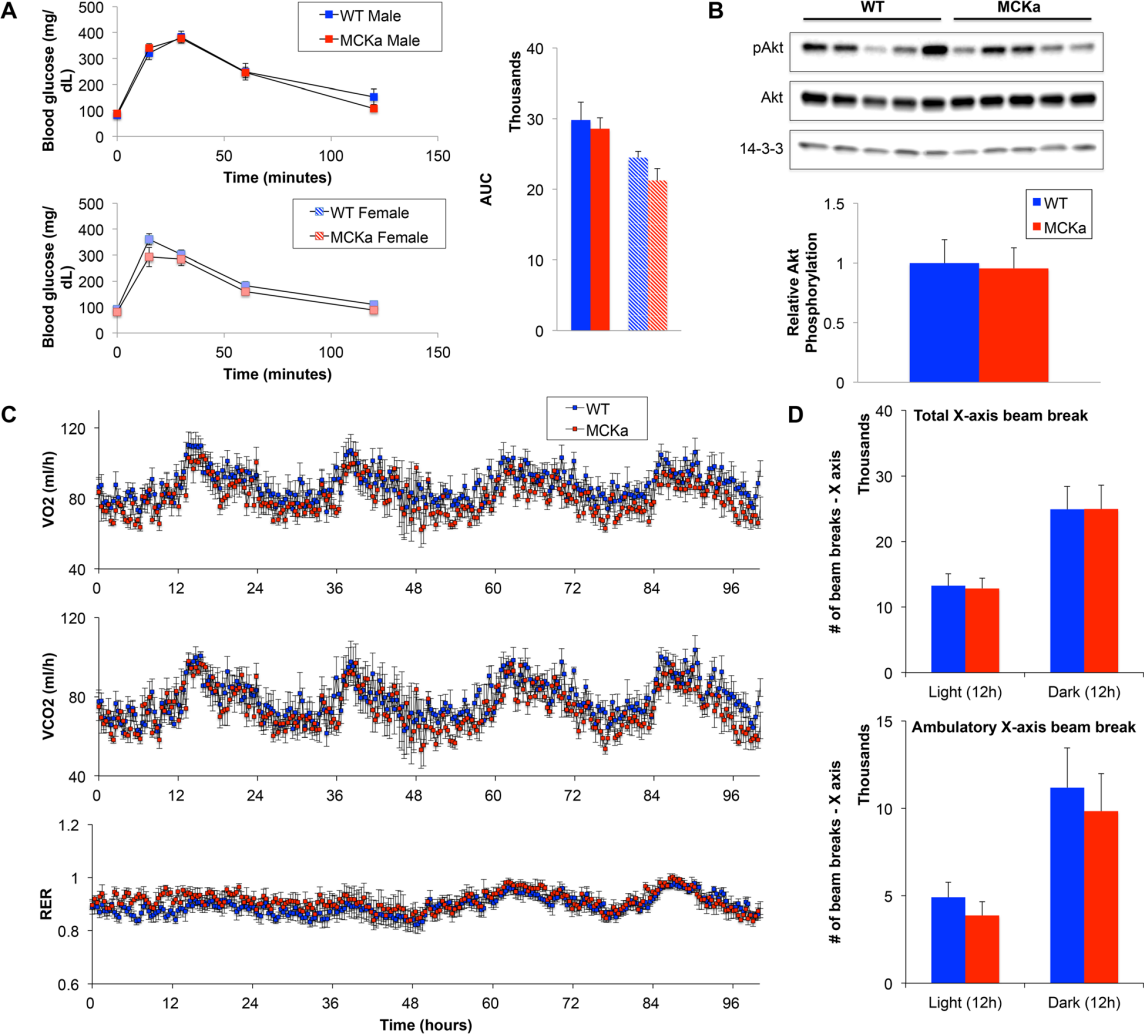
Skeletal muscle PGC-1alpha has little effect on whole-body metabolism in 24-month-old mice. **a** Glucose tolerance test (GTT) in male and female 24-month-old mice. Calculated AUC of GTTs. **b** Western blots for pAkt and Akt in quadriceps (quantification is normalized to loading control 14-3-3). **c** VO2, VCO2, and respiratory quotient (RER) in 24-month-old mice measured via Comprehensive Laboratory Animal Monitoring System (CLAMS). **d** Physical activity of 24-month-old mice measured via CLAMS [*n* = 5, 5 for 24-month-old WT and MCKa]. *P < 0.05 compared to control

Finally, PGC-1alpha in skeletal muscle has been reported to variably affect whole-body metabolism in young animals [23, 24]. We thus used a glucose tolerance test to evaluate the impact of muscle PGC-1alpha overexpression on insulin resistance in aged animals. Transgenic and wildtype littermate control animals showed similar glucose excursions, and similar kinetics of glucose clearance, after glucose bolus (Fig. 5a). Western blot analysis revealed no alterations in Akt phosphorylation in muscle from aged MCKa animals compared to controls (Fig. 5b). Finally, evaluation with Comprehensive Laboratory Animal Monitoring System (CLAMS) revealed no difference in oxygen consumption (Fig. 5c), respiratory quotient (RER), circadian rhythm, or physical activity (Fig. 5d) between the transgenic and littermate control animals. We conclude that PGC-1alpha induction in skeletal muscle has little impact on whole body metabolism in old age.

## Discussion

We show here that transgenic expression of PGC-1alpha in skeletal muscle, to an extent comparable with that induced by exercise [8–10], leads to multiple effects in aged animals. The most striking of these is the continued induction of both the mitochondrial and angiogenic programs. Thus, these data demonstrate that aged skeletal muscle remains susceptible to the effects of PGC-1alpha, an important observation if PGC-1alpha is to be considered as a therapeutic target in aging. The data similarly demonstrate that angiogenic and mitochondrial biogenic programs remain capable of being activated in aged animals, also an important observation when considering activating these pathways in aging.

Equally striking and potentially of clinical interest is the dramatic impact of PGC-1alpha expression on aged muscle contractility. The marked reduction in muscle fatigability is most likely due to the mitochondrial expansion and increased oxidative capacity, and less likely due to angiogenesis, since the phenotype is pronounced even in isolated muscles. Of note, however, the benefits on fatigability are gained at the expense of overall twitch force. This loss does not simply reflect the smaller oxidative fibers or small muscles, because force was normalized to muscle cross-sectional area. Activation of PGC-1alpha pathways in aged individuals may therefore have undesired effects, since loss of strength is an important component of frailty.

The observed effects of PGC-1alpha on aged bone density would similarly be unfavorable in the clinic. Loss of trabecular bone, as was seen in the transgenic animals, is a hallmark of age-associated bone loss [19, 20]. Consistent with this, we find a strong trend of MCKa bones to early fracture under a bend test. These findings are at odds with those reported by the retracted 2009 paper [17]. Interestingly, cortical bone was increased in male MCKa mice. The reason for this increase is not clear, but may be related to their higher body weight, and the known relationship between lean mass and bone density. Alternatively, signals elicited from the transgenic muscle may have opposing effects on cortical and trabecular bone. For example, we find significant induction of mRNA expression of the PGC-1alpha target *Fndc5* in aged MCKa animals, and injection of cleaved FNDC5 (aka irisin) in mice for 4 weeks has been shown to improve cortical bone mass [25], while conversely, genetic deletion of FNDC5 was recently reported to block osteolysis induced by ovariectomy, specifically affecting trabecular, and not cortical bone [21]. It may thus be that transgenic induction of PGC-1alpha in aged muscle promotes specifically trabecular bone loss via the induction and secretion of FNDC5, acting in either a paracrine or endocrine fashion. We cannot rule out, of course, that secreted factors other than FNDC5, or neural circuits, may contribute to the loss of bone density. It is also worth noting that individuals who regularly perform aerobic exercise, in whom FNDC5 is presumably induced, are in fact protected against bone loss, both trabecular and cortical. The MCKa mice thus likely only model a subset of the effects of aerobic exercise.

The worsening of age-associated bone loss is not the only finding that differs from those reported in the retracted 2009 paper. Our results also reveal no clear benefits on whole-body metabolic homeostasis of lifelong muscle overexpression of PGC-1alpha. We recognize the statistical limitations of our small cohorts, necessitated by the length of the studies. Nevertheless, overall, our findings suggest that therapeutic induction of PGC-1alpha in aged skeletal muscle may, on balance, be contraindicated.

## Conclusion

Sustained PGC-1alpha activation in mouse skeletal muscle into old age benefits muscle fatigability and may protect from sarcopenia in males, but does not improve whole-body metabolism, and worsens age-related trabecular bone loss. Our findings suggest that therapeutic induction of PGC-1alpha in aged skeletal muscle may, on balance, be contraindicated.

## Data Availability

All data generated or analyzed during this study are included in this published article.

## Abbreviations

PGC-1alpha: Peroxisome-proliferator-activated receptor-γ coactivator-1 alpha
MCK: Muscle creatine kinase promoter
MCKa mice: MCK-PGC-1alpha transgenic mice
qPCR: Quantitative real-time polymerase chain reaction
OXPHOS: Oxidative phosphorylation
CD31: Cluster of differentiation 31
EDL: Extensor digitorum longus
CLAMS: Comprehensive Laboratory Animal Monitoring System
RER: Respiratory quotient
FNDC5: Fibronectin Type III Domain Containing 5
